# Solid-State Study of the Structure, Dynamics, and Thermal Processes of Safinamide Mesylate—A New Generation Drug for the Treatment of Neurodegenerative Diseases

**DOI:** 10.1021/acs.molpharmaceut.1c00779

**Published:** 2021-12-03

**Authors:** Tomasz Pawlak, Marcin Oszajca, Małgorzata Szczesio, Marek J. Potrzebowski

**Affiliations:** † Centre of Molecular and Macromolecular Studies, Polish Academy of Sciences, Sienkiewicza 112, 90-363 Lodz, Poland; ‡ Faculty of Chemistry, Jagiellonian University, Gronostajowa 2, 30-387 Krakow, Poland; § Institute of General and Ecological Chemistry, Faculty of Chemistry, Lodz University of Technology, Żeromskiego 116, 90-924 Lodz, Poland

**Keywords:** Parkinson disease, Xadago, DFT-D, GIPAW, very-fast MAS NMR, disorder structures, quantum mechanics calculations, API, Phase transition, molecular dynamics

## Abstract

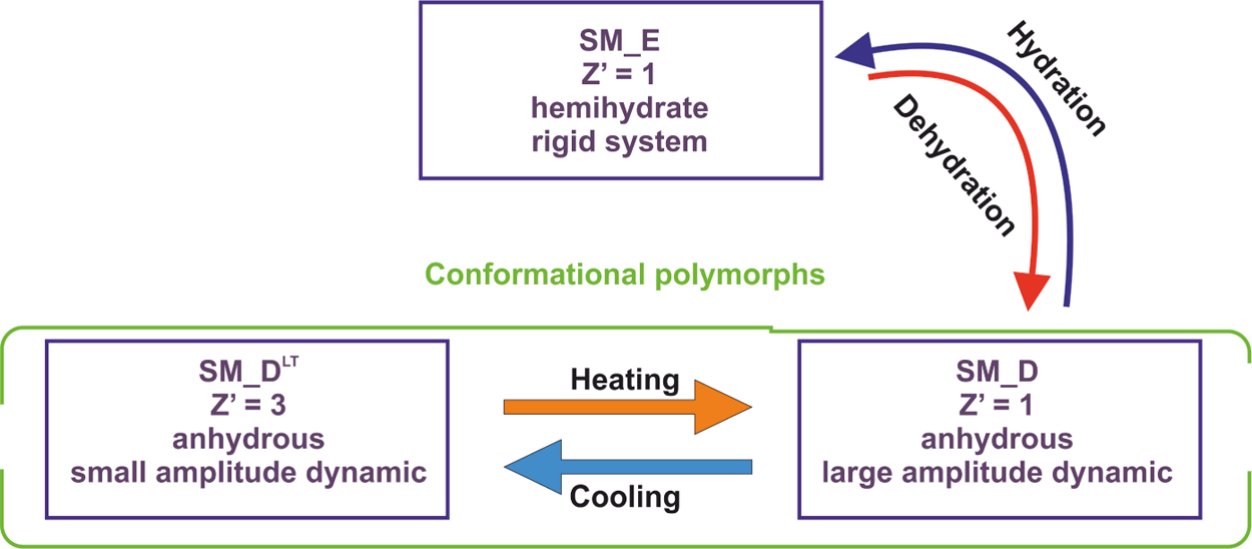

Safinamide mesylate (**SM**), the pure active pharmaceutical ingredient (API) recently used in Parkinson disease treatment, recrystallized employing water–ethanol mixture of solvents (vol/vol 1:9) gives a different crystallographic form compared to **SM** in Xadago tablets. Pure **SM** crystallizes as a hemihydrate in the monoclinic system with the *P*2_1_ space group. Its crystal and molecular structure were determined by means of cryo X-ray crystallography at 100 K. **SM** in the Xadago tablet exists in anhydrous form in the orthorhombic crystallographic system with the *P*2_1_2_1_2_1_ space group. The water migration and thermal processes in the crystal lattice were monitored by solid-state NMR spectroscopy, differential scanning calorimetry, and thermogravimetric analysis. **SM** in Xadago in the high-humidity environment undergoes phase transformation to the *P*2_1_ form which can be easily reversed just by heating up to 80 °C. For the commercial form of the API, there is also a reversible thermal transformation observed between *Z*′ = 1 ↔ *Z*′ = 3 crystallographic forms in the 0–20 °C temperature range. Analysis of molecular motion in the crystal lattice proves that the observed conformational polymorphism is forced by intramolecular dynamics. All above-mentioned processes were analyzed and described employing the NMR crystallography approach with the support of advanced theoretical calculations.

## Introduction

**Figure 1:**
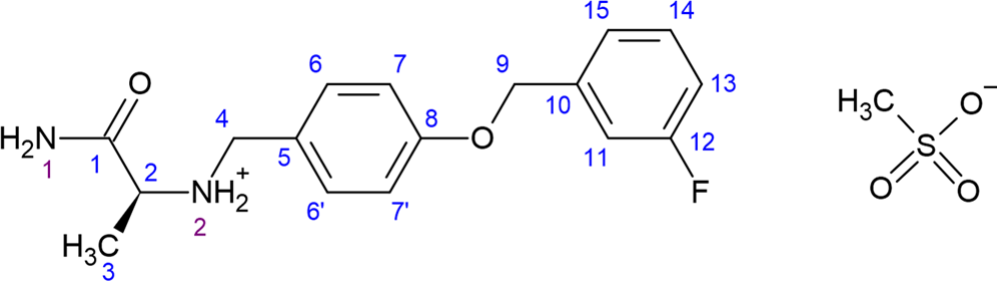
Chemical structure of **SM** and numbering system.

Neurodegenerative diseases affect millions of people worldwide.^1,2^ With increasing global population and average lifespan, the prevalence of neurological disorders is on the rise. The risk of being affected by a neurodegenerative disease increases dramatically with age. In developed countries, life expectancy is now rising well above 80 years.^3,4^ Although in older people, the prevailing death causes are still cardiovascular diseases and cancer, Alzheimer’s disease, Parkinson’s disease (PD), amyotrophic lateral sclerosis, and other neurodegenerative disorders are known to be strongly age-related.^5,6^ Among the 10 top illnesses ending with death, neurodegenerative diseases cannot yet be fully cured or slowed down. Therefore, it is not surprising that a great deal of effort goes into finding new medicines to treat neurodegenerative diseases. Very recently, a new drug under the Xadago brand name has been introduced in the pharmaceutical market for the treatment of PD’s disease.^7,8^ The active pharmaceutical ingredient (API) of Xadago is safinamide mesylate (**SM**) salt (molecular structure shown schematically in Figure 1).^9,10^

The mechanism of the drug action operates through the inhibition of monoamino oxidase-B, an enzyme responsible for the breakdown of dopamine. As a result, an increase of the dopamine level in the brain for subsequent dopaminergic activity in PD patients is observed. Moreover, safinamide shows nondopaminergic actions such as sodium channel blocking and inhibition of glutamate release.^11^ Xadago was approved in Europe in February 2015, in the United States in March 2017, and in Canada in January 2019. This drug is administered orally in the form of solid tablets.

Despite very advanced clinical studies, little is known about the solid-state properties of **SM**, both as a pure API and as a tablet component. Although an international patent describing the three crystallographic forms of **SM** has been known for many years, to the best of our knowledge, there are no detailed studies showing the complexity of this system.^12^ Very recently, Nanubolu has reported in-depth X-ray studies of safinamide acid hydrochloride.^13^ Two concomitant polymorphs were obtained in an attempt to prepare the O-protonated amide salt of safinamide from ethanolic HCl solution. Polymorph I crystallized in the triclinic space group *P*1 with three molecules in the asymmetric unit (*Z*′ = 3), while polymorph II crystallized in the orthorhombic space group *P*2_1_2_1_2_1_ with a single molecule in the asymmetric unit (*Z*′ = 1). The high *Z*′ = 3 structure showed a phase transition to a *Z*′ = 2 structure in a single-crystal to single-crystal fashion. In contrast, the *Z*′ = 1 polymorph I did not show any such phase transition.

Polymorphism and thermal stability of an API are the most important properties that determine the usefulness of drugs in therapeutic treatment.^14−16^ Polymorphism is the ability of the substance to crystallize in more than one crystalline phase with different arrangements or conformations of the molecules in the crystal lattice.^17^ Over 50% APIs are estimated to have more than one polymorphic form. It is well known that polymorphs differ in physical properties such as molecular packing, melting point, fusion enthalpy, dissolution behavior, and bioavailability.^18,19^ API polymorphs can also exhibit different physical and mechanical properties, including hygroscopicity, particle shape, density, flowability, and compactibility,^20−22^ which can affect the processing of manufacturing of the drug product and require control over all stages of synthesis, application, and storage.^23,24^ The most important issue in the research of API polymorphs is to identify their properties as part of the quality assurance process. To find the best form of the drug, many different advanced characterization techniques should be used. The ultimate goal is to select the most thermodynamically stable form to be able to manufacture it consistently. An incident involving the anti-HIV/AIDS drug ritonavir highlighted the need for greater control of the drug‘s polymorphism and prompted companies and scientists to undertake the comprehensive screening of polymorphous modifications.^25^ Since the goal of finding polymorphs with the most desirable properties is not easily accessible, works in this area are very important.

The facts mentioned above and the general knowledge about drug polymorphism prompted us to deal with Xadago tablets in detail employing advanced instrumental techniques [solid-state NMR spectroscopy, single-crystal X-ray diffraction, powder X-ray diffraction (PXRD), differential scanning calorimetry (DSC), and thermogravimetric analysis (TGA)] and theoretical approaches.

## Experimental Procedures

### Obtaining of Starting Materials

Xadago is a commercially available product. For the purpose of this study, it was purchased from a pharmacy. The production serial number was 9517442106126 with the expiration date of 03/2024.

### Single-Crystal X-ray Measurements

The X-ray data were collected on a diffractometer (XtaLAB Synergy, Dualflex, Pilatus 300K, Rigaku Corporation, Tokyo, Japan) at 100 K with a microsource of Cu-Kα radiation (λ = 1.5418 Å) and a Titan detector (Oxford Diffraction, Agilent Technologies, Yarnton, U. K.) equipped with an 800 Cryostream low-temperature unit (Oxford Cryosystems, Oxford, U.K.).

Diffraction data collection, cell refinement, data reduction, and absorption correction were performed using CrysAlis PRO software (Agilent Technologies UK Ltd., Yarnton, England). Structures were solved by the direct method SHELXS^26^ and then refined using the full-matrix least-squares method SHELXL 2015^27^ implemented in the OLEX2 package.^28^ In all of the crystal structures, the non-hydrogen atoms were present in the direct method solution.

### PXRD Measurements

PANalytical X’Pert PRO MPD powder diffractometer was used in the collection of diffraction data on the powder samples. The instrument was equipped with a sealed LFF X-ray tube with a copper anode, an elliptic X-ray focusing mirror, and a PIXCEL detector. Divergence slit of 1/2° and 0.02 rad. Soller slits (in both incident and diffracted beam paths) were applied. The powder sample was packed inside a 0.7 mm diameter Hilgenberg borosilicate glass capillary and measured in a repeated scan mode during a four scan measurement. The registered data range was 3–85° 2θ with a step of 0.02°, and the collection time setting made each scan last 3.5 h. The obtained scans were tested for any discrepancies suggesting adverse reaction of the sample to X-ray irradiation and summed up.

The experimental diffraction data were indexed in an orthorhombic cell applying the successive dichotomy method DICVOL04 as implemented in Expo2014 software.^29−31^ The global optimization technique using FOX^32,33^ with the application of a safinamide and methanesulfonate molecule model was applied. Multiple runs of calculations were performed with 8 × 10^6^ trials per run, and the best obtained solution was selected based on the smallest data fitting discrepancies, as well as the general sense of the calculated model was chosen for the structure refinement stage. Rietveld method implemented in GSAS-II was used in the refinement step.^34^ At the final fitting, 15 Chebyshev points were used to describe the background. The refinement of the non-hydrogen atomic positions was carried out with bonds and angles restraints based on Mogul CCDC parameters.^35,36^

### NMR Spectroscopy

Cross-polarization magic-angle spinning (CP MAS) NMR, one-pulse ^1^H MAS, and polarization inversion spin exchange at the magic angle (PISEMA) MAS^37−39^ experiments were performed on a 400 MHz Bruker AVANCE III spectrometer operating at 400.15, 100.62, and 40.55 MHz for ^1^H, ^13^C, and ^15^N, respectively, equipped with a HX MAS probe head using 4 mm rotors.

A sample of U–^13^C, ^15^N-labeled histidine hydrochloride was used to set the Hartmann–Hahn condition for ^13^C and ^15^N. ^1^H → ^13^C and ^1^H → ^15^N CP MAS experiments on the 400 MHz Avance III spectrometer were performed at a MAS frequency of 8 kHz with a proton 90° pulse length of 4 μs and a contact time of 2 ms for ^13^C and 8 ms for ^15^N. For CP, the nutation frequency was 54.5 kHz for ^13^C as well as for ^15^N with a ^1^H ramp shape from 90 to 100% with a ^1^H nutation frequency of 62.5 kHz. For ^13^C and ^15^N, 3.5k and 2k data points were acquired for a spectral width of 40 and 28 kHz, respectively. In all cases, SPINAL-64 decoupling sequence^40^ with a ^1^H nutation frequency of 71.4 kHz and a pulse length of 7 μs were applied (also for the PISEMA experiment described below).

The PISEMA MAS experiment^37−39^ was carried out with an ^1^H nutation frequency of 82.5 kHz in all of the experiments, and the ^13^C spin-lock field strengths were adjusted to the first-order sideband condition, ω_13C_ = ω_1H_ ± ω_r_. The spinning frequency was 13 kHz and was regulated to ±3 Hz by a pneumatic control unit. 256 coadded transients for each of 64 *t*_1_ FIDs correspond to a total experimental time at 23 h. The 2D PISEMA MAS experiments incremented the SEMA contact time using a step of 16.28 μs, with a maximum *t*_1_ evolution time of approximately 1 ms. Since the *t*_1_ time signal increases with increasing SEMA contact time, the ω_1_ dimension was processed using the baseline correction mode “qfil” in Bruker TopSpin 3.5 program software,^41^ which subtracted a constant intensity from the time signals prior to the Fourier transformation and yielded spectra free from the dominant zero-frequency peak that gives the ^1^H–^13^C doublet.

Fast MAS spectra were recorded on a 600 MHz Bruker Avance III spectrometer operating at 600.13 and 150.90 MHz for ^1^H and ^13^C, respectively, equipped with a HCN MAS probe head operating in the double-resonance mode using 1.3 mm ZrO_2_ rotors with a spin rate of 60 kHz. The ^13^C–^1^H-invHETCOR experiments were performed using the pulse sequence described elsewhere.^42−44^ The following parameters were used: a proton 90° pulse length of 2.5 μs and a first and second contact time of 2 ms and 100 100 μs, respectively, both with a ^1^H 90–100% ramp shape. The ^1^H and ^13^C nutation frequency was 160 and 109 kHz, respectively, for both CP steps. The acquisition data were collected with a SWf-TPPM^45,46^ decoupling sequence with a ^1^H nutation frequency of 10 kHz and a pulse length of 50 μs.^40^ The States-time-proportional phase incrementation method was employed for sign discrimination.^47^

The ^13^C chemical shift was referenced indirectly by using adamantane (resonances at 38.48 and 29.46 ppm) as an external secondary reference.^48,49^ The ^15^N glycine (resonances at 34.40 ppm) was used as a secondary chemical shift reference for ^15^N.^49,50^ The real temperature inside the MAS rotor is different from the ambient temperature, mostly due to frictional effects caused by rotor spinning.^51^ Because of that, Pb(NO_3_)_2_ was used for temperature calibration.^52^ Except where otherwise stated, a recycle delay of 5 s was used.

### Quantum Mechanics Calculations

Density functional theory (DFT) calculations were performed with periodic boundary conditions using the CASTEP 19.11 code.^53^ The geometry optimization was performed using the X-ray diffraction crystal structures as an input file by varying all atoms and the unit cell parameters. The geometry optimizations were performed until the energy converged to within 10^–7^ eV. The generalized density approximation DFT functional Perdew–Burke–Ernzerhof with the TS dispersion correction scheme (DFT-D method) was applied.^54,55^ A comparison of the average forces remaining on the atoms after geometry optimization with a convergence limit of 0.02 eV/Å was carried out by using a maximum plane wave cutoff energy of 620 eV and an ultrasoft pseudopotential.^56^ The optimization algorithm was BFSG,^57^ and the Monkhorst–Pack grid^58^ of minimum sample spacing 0.07 × 2π Å^–1^ was used to sample the Brillouin zone. The NMR chemical shifts were computed using the gauge-including projected augmented wave (GIPAW) method.^53,59,60^ The calculated NMR chemical shieldings were converted into chemical shifts by linear regression between calculated and experimental results.

### Other Methods (DSC, TGA, Elemental Analysis)

DSC and TGA were recorded using a DSC 2920 (TA Instruments) calorimeter with the heating rate of 5 °C min^–1^. Elemental analysis of hydrogen, carbon, and nitrogen was performed using CE Instruments.

## Results and Discussion

### PXRD and Solid-State NMR Analysis of Xadago Tablet

**Figure 2:**
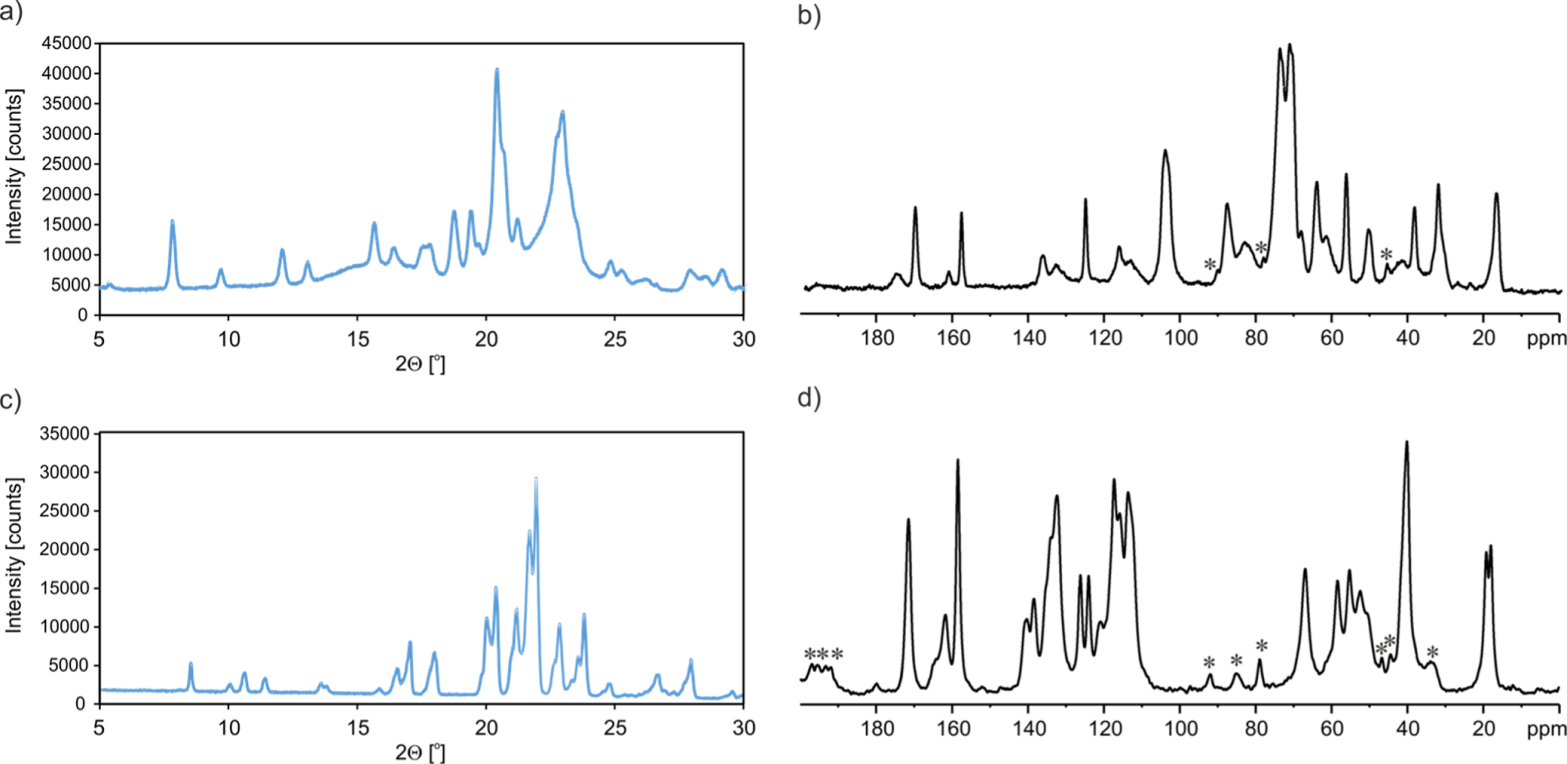
Results for the Xadago drug (a,b) and API extracted from tablets (c,d) at room temperature: (a,c) X-ray powder pattern recorded in Bragg Brentano geometry with a Cu Kα (λ = 1.5425 Å) source. (b,d) ^13^C CP MAS NMR spectrum of the Xadago drug recorded at a spinning rate of 8 kHz and a ^1^H Larmor frequency of 400.1 MHz. The recycle delay was 30 s. Asterisks indicate spinning sidebands.

We began our study with PXRD and ^13^C CP MAS NMR measurements carried out at ambient temperature for a commercially available Xadago tablet (Figure 2). Both techniques clearly proved that the tablet contains **SM** in the crystalline form. However, apart from the high-crystalline components, there are also much broader reflexes (Figure 2a) or signals (Figure 2b) visible, reflecting the amorphous background (Figure 2a). It is not surprising since the drug formulation usually contains various substances that support the manufacturing process. According to the product characteristic declaration, the Xadago drug apart from the **SM** substance contains a series of following excipients: cellulose, polyvidone, magnesium stearate, and colloidal anhydrous silica.^10^ These additional components complicate the precise analysis of the crystalline form of **SM**, and hence in the next step, we decided to extract the pure API from the tablet.

It was possible to isolate pure **SM** by fast filtering-off the insoluble components using water as a solvent. Next, the API was crystallized from water solution by isothermic evaporation. Figure 2c,d shows the X-ray powder pattern and the ^13^C CP MAS spectrum of the extracted API. As one can see, both measurements prove that the obtained sample is pure, homogeneous, very well organized, and crystalline without the large amorphous background visible in the X-ray powder pattern of the Xadago tablet (Figure 2a). At this point, the most thought-provoking observation was the mismatch for the reflection positions (PXRD) as well as the signal positions (solid-state NMR) between the Xadago tablet and the extracted API material. The first, simple explanation of these discrepancies is based on the assumption that we observe different polymorphs of **SM**. The detailed explanation of that will be presented in the following sections.

### X-ray Determination of the SM_E Single-Crystal X-ray Structure

**Figure 3:**
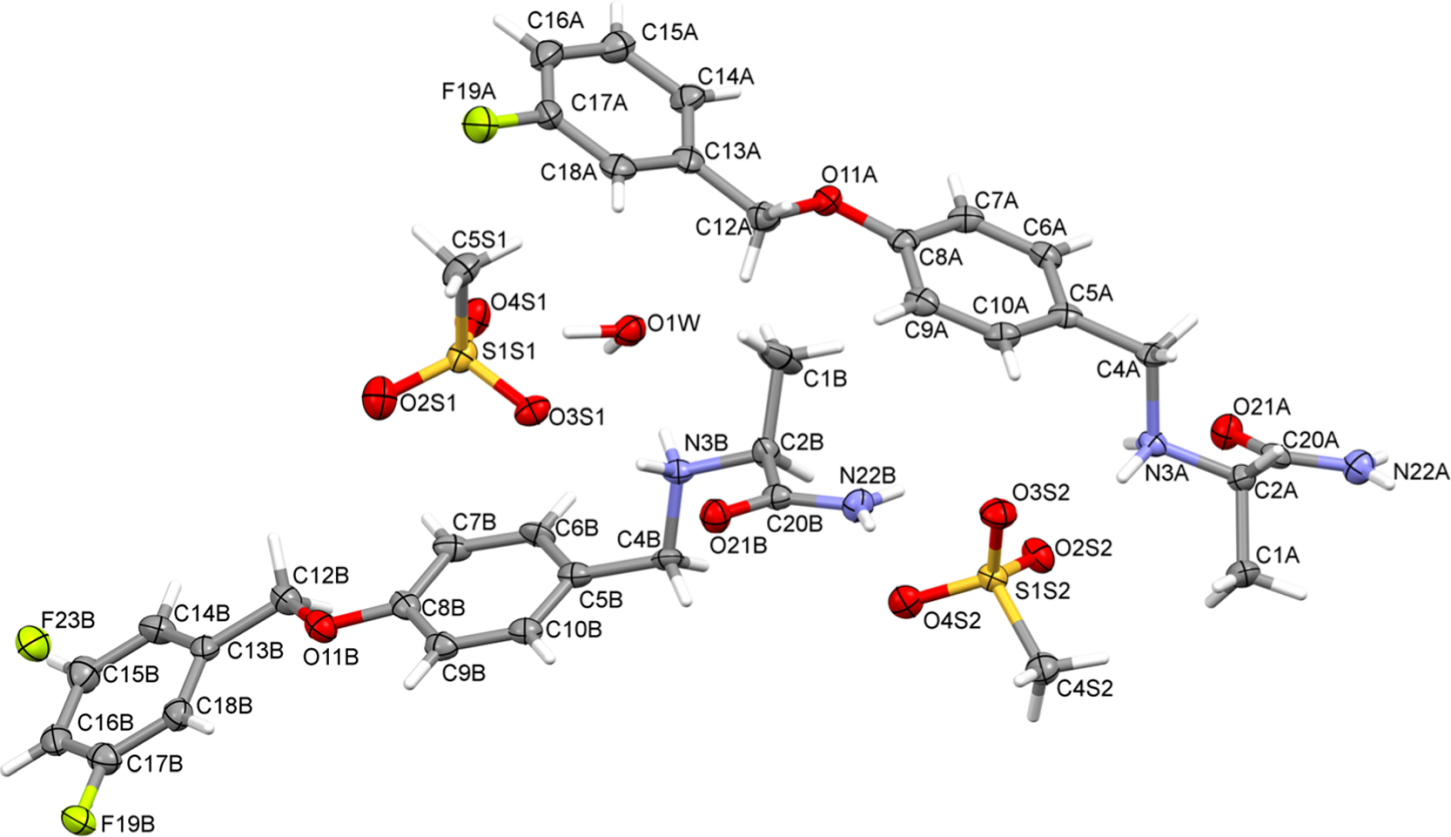
Asymmetric part of the unit cell of **SM_E** showing the crystallographic atom-labeling scheme. Displacement ellipsoids are drawn at the 50% probability level except for the H atoms.

In order to test the susceptibility of **SM** to form different polymorphs, the API sample was recrystallized from various solvents belonging to the Generally Recognized as Safe FDA list.^61^ After several attempts, the best quality material allowing to determine the crystal structure from single-crystal X-ray diffraction data was obtained by the recrystallization of API from water/ethanol (1:9) solution (further referred to as **SM_E** form). The structure deposited in CCDC under no. 1899715 is shown in Figure 3. The corresponding crystallographic data are presented in Table 1.

**Table 1:** Crystal Structure and Refinement Data for **SM_E**

empirical formula	2(C_17_H_20_FN_2_O_2_)·2(CH_3_O_3_S)·H2O
formula weight	814.90
temperature	100 K
crystal system	monoclinic
space group	*P*2_1_
*a* (Å)	5.6001 (3)
*b* (Å)	20.4399 (9)
*c* (Å)	16.7486 (9)
α (deg)	95.839 (4)
volume (Å)^3^	1907.19 (17)
*Z*	4
*Z*′	2
*R*-factor (%)	7.67
no. of measured, independent and observed [*I* > 2*s*(*I*)] reflections	13021, 6368, 5527
*R*_int_	0.072
*R*[*F*2 > 2*s*(*F*^2^)], wR(*F*^2^), *S*	0.077, 0.210, 1.10

**Figure 4:**
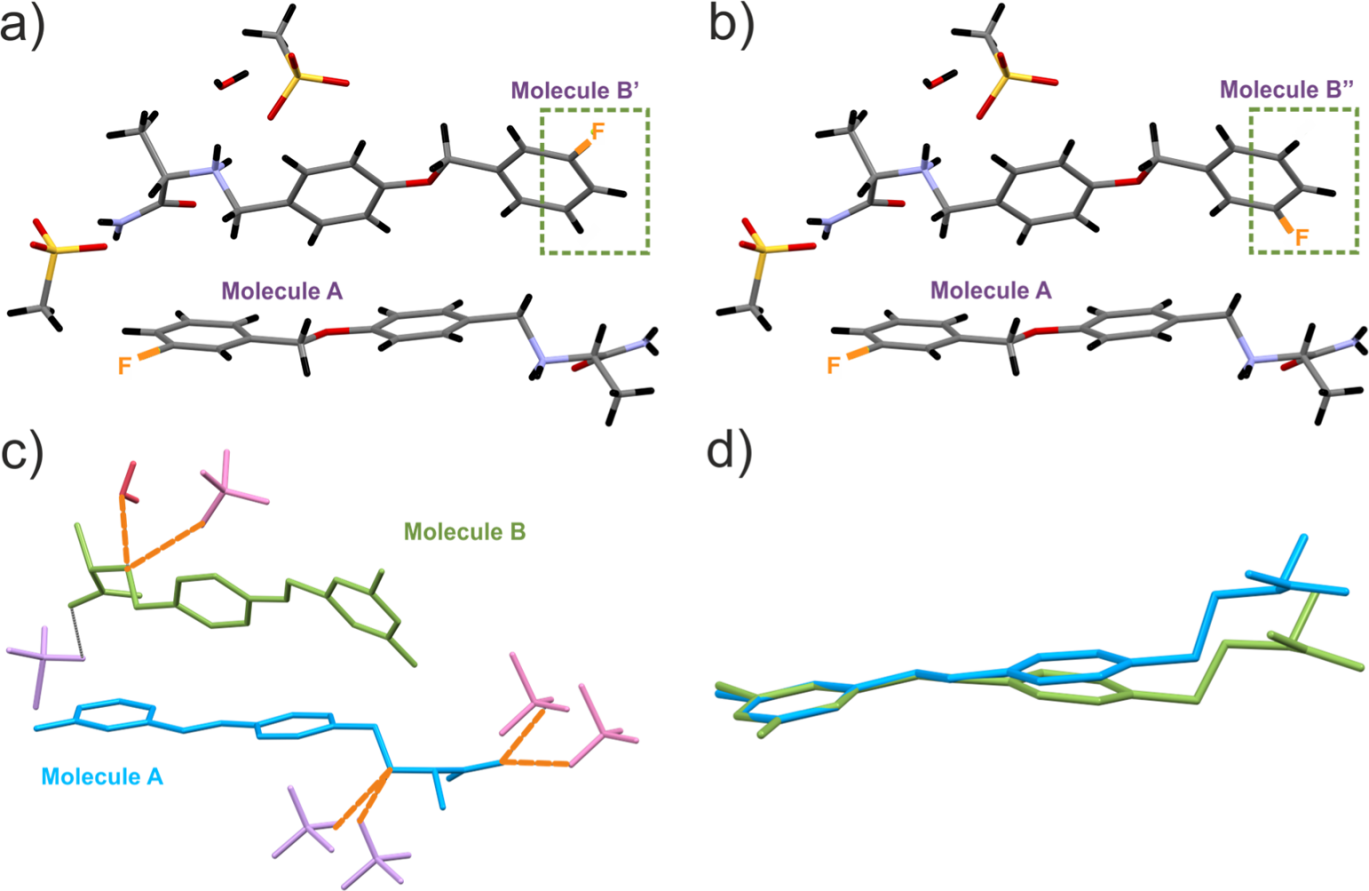
Crystal structure of **SM_E** indicating (a,b) asymmetric parts of the unit cell with two possible locations of F atoms (fractionally occupied) outlined by dotted green lines, (c) hydrogen bond motifs (colored orange), and (d) superposition of nonequivalent molecules A and B extracted from the single-crystal diffraction-based structure of **SM_E**. Molecules (c,d) are colored according to symmetry equivalence and shown without hydrogens (except for the water molecule).

Up to date, only two safinamide polymorphs are known (refcodes TUWFIB and TUWFIB01),^62^ and no other crystal structures containing safinamide have been deposited in Cambridge Structural Database (CSD).^63^ The **SM_E** form obtained in our work was also reported in the patent claim as form **H1**, though there are no structural details provided except for the unit cell dimensions and positions of powder pattern reflections.^12^ Even a brief look at the structure of the **SM_E** crystal shows that the introduction of the mesylate anion significantly changes the organization of the crystal lattice compared to the safinamide polymorphs TOWFIQ and TUWF IB01.^62^ The **SM_E** crystallizes under the *P*2_1_ symmetry with 1905.0 Å^3^ volume. There are two independent safinamide molecules (further referred to as “A” and “B”) in the crystal lattice exhibiting different hydrogen-bonding motifs. The unit cell also contains an equivalent number of mesylate anions and a water molecule per two safinamide molecules (Figure 4a) which classifies the structure as a hemihydrate. The first visible geometrical difference between A and B molecules is the position of the aromatic ring containing the fluorine atom. For molecule B, the fluorinated phenyl ring (or more specifically the fluorine atom) occupies two positions, whereas it is not the case for molecule A. In this way, two alternative conformations of the B molecule arise, which we will denote in the text as B′ and B″ and refer their names to the safinamide molecules as presented in Figure 4a,b. There are two theoretically justified variants of the rigid structure most probably connected with either positional disorder or molecular dynamics of the phenyl ring with a jump angle of 180°. The second important feature is connected with different contacts (hydrogen bonds) between molecules A and B (Figure 4b). As it is clearly seen, one of the safinamide molecules (colored blue) does not conjugate with water, whereas the other (colored green) has such an interaction. The last important difference can be easily recognized by making a superposition of both nonequivalent safinamide molecules (Figure 4d). The main difference comes from the C8–O–C9–C10 torsional angle (differing by ∼10°) in both forms. These observations will be confronted later in the text with solid-state MAS NMR results.

**Figure 5:**
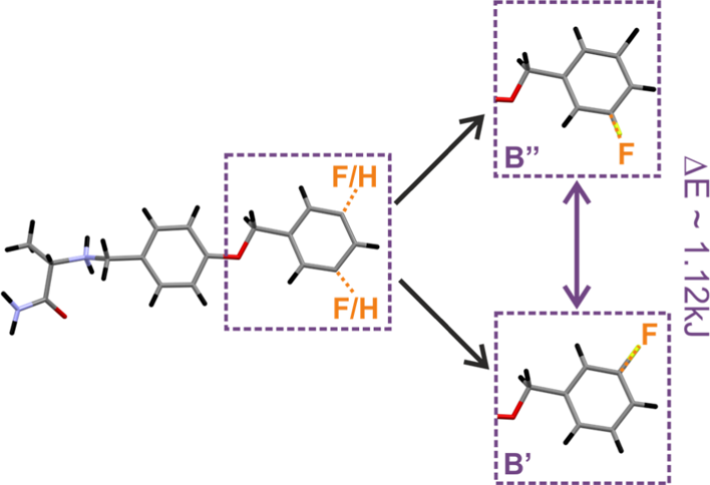
Schematic representation of two possible orientations of fluorine in molecule B and the relative total crystal lattice energy difference at the DFT-D level between structures containing B′ and B′ conformers.

Keeping in mind that the structure of **SM_E** presents an uncertain location of the fluorine atom, we investigated the nature of this feature and pointed out the most plausible orientation of the fluorophenyl ring. At this stage, we constructed two B′ and B″ theoretical models with a different location of the fluorine atom in molecule B and performed the DFT-D optimization of all atomic positions as well as unit cell parameters for them. The obtained structures have less than 1% difference in the unit cell parameters and extreme similarity of atomic positions except for the fluorine atom (see the attached crystallographic structure after DFT-D calculations in the Supporting Information). Figure 5 shows the energy difference between the two models in pictorial form. The total crystal lattice energy differs between structures containing orientations B′ and B″ only by about 1.12 kJ. It means that B′, where the fluorine atom is “trans” with respect to oxygen, is slightly more preferred. It is an extremely small value, and it suggests that the orientation of the phenyl ring may be easily inverse to the opposite conformation at the room temperature. Note that such DFT calculations only probe the thermodynamics (i.e., equivalent to 0 K), and temperature-dependent kinetic effects are not considered. Apart from the above, the inspection of close contacts and free volume analysis in the unit cell using Mercury software^64,65^ do not support the possibility of reorientation process because the packing in the crystal lattice forbids it. It means that the observed feature has to be determined during the crystallization process and later remains unchanged. The absence of a determining energetic preference and steric effect is consistent with nearly 50% occupancy of the fluorine atom in both refined positions. In that way, such an observation is a clear example of a static disorder.

### Validation of SM_E X-ray Structure by Means of Advanced Solid-State NMR and DFT-D Calculations. Analysis of ^13^C and ^15^N Chemical Shifts

As the first point of the validation of the **SM_E** structure, we applied advanced NMR methodologies for precise assignment of the ^13^C and ^15^N chemical shifts. Based on the liquid-state NMR measurements, we attempted to assign NMR signals in the solid state. Although it might be a valid strategy to obtain a first approximation, the large number of observed resonances and their insufficient dispersion in ^13^C CP MAS spectra for more complicated cases cause the need to apply a more advanced methodology. It should be noted that the position and assignment of signals for solid-state and liquid-state NMR can change, especially for peaks which are very close to one other.^66,67^ Therefore, the uncritical cloning of the liquid-state assignments to the solid-state spectra should not be attempted at all. In our case, we decided to apply theoretical calculations and compare them with the experimental data. The details regarding the computations and experimental methods will be discussed later in this chapter.

**Figure 6:**
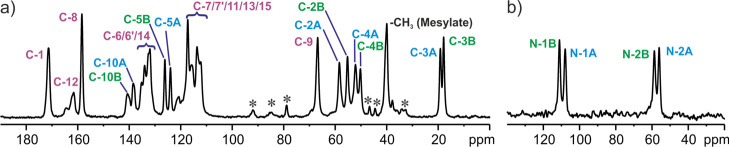
^13^C (a) and ^15^N (b) CP MAS NMR spectra of **SM_E** recorded at a spinning rate of 8 kHz and a ^1^H Larmor frequency of 400.1 MHz at ambient temperature. Assignments are colored blue for molecule “A”, green for molecule “B,” and purple for overlapped signals. Asterisks indicate spinning sidebands.

**Figure 7:**
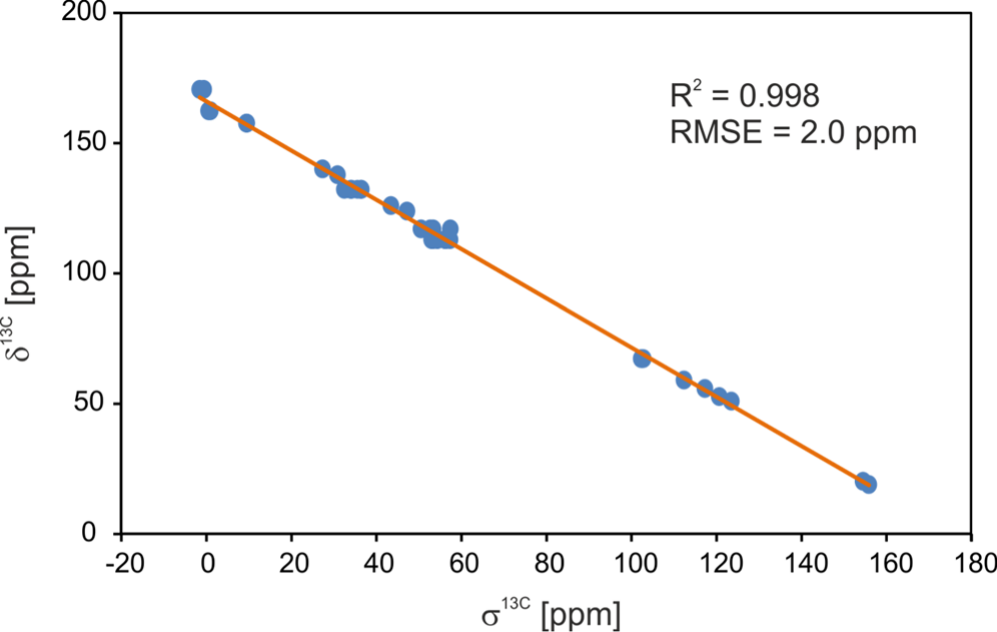
Correlation of experimental ^13^C chemical shift (δ) and calculated nuclear shielding parameters (σ) of **SM_E**.

The ^13^C and ^15^N CP MAS spectra of **SM_E** are shown in Figure 6. As it was described in the previous section, the **SM_E** structure contains two crystallographically nonequivalent molecules in the asymmetric part of the unit cell. However, even a brief look at the ^13^C spectrum shows that most of the positions are isochronous and overlap giving single resonances. Moreover, the differences between those which are separated are not very large and constitute up to 3.5 ppm. The image of ^13^C as well as ^15^N CP MAS spectra confirms without a shadow of a doubt the presence of two nonequivalent molecules in the asymmetric part of the unit cell. The full assignment of ^13^C and ^15^N signals to the conformers “A” and “B” is not straightforward. To solve this problem, we applied the GIPAW method,^33−35^ which constitutes a breakthrough in the theoretical prediction of NMR parameters for solid materials. This approach has been used in many spectacular applications, and its usefulness in the spectral analysis is unquestionable.^68−81^ Using the GIPAW strategy, the signal assignment was made by comparing the experimental values of chemical shifts and those calculated theoretically. The final result is shown in Figure 7. Excellent agreement is observed between experimental and calculated ^13^C chemical shifts (GIPAW), as reflected by the small root-mean-squared error (RMSE) values of 2.0 ppm (Table S1).^72,82−86^ Using the machine learning method, Emsley et al. established the RMSE threshold for organic molecules at the level of 4.3 ppm for ^13^C, which defines the correctness of the structure solution.^87^ Our results (RMSE equal 2) clearly prove that the selected monocrystal for X-ray measurement is representative of the bulk material.

The colors used to label the NMR signal (Figure 6) match the colors of the structure shown in Figure 4c (purple indicates overlapping signals). If we compare the NMR results with the structure shown in Figure 4c, it is obvious that the positions for which we observed the main geometrical discrepancies between the molecules “A” and “B” are also recognized as magnetically nonequivalent in the NMR spectrum. ^13^C CP MAS measurements, often used in pharmaceutical sciences^39,50,56−76^ for comparative drug analysis, show that **SM_E** and API material extracted from a tablet using the procedure described in section (i) represent a similar crystallographic form, but the crystallinity of **SM_E** is much higher. It is worth recalling that according to PXRD and ^13^C CP MAS measurements in Xadago tablets, we observed a different form of API. This inconsistency prompted us to undertake further studies.

### Thermal Transformations of Sample SM_E

In the course of our studies, we observed a broadening of the NMR signals and a change of the ^13^C CP MAS **SM_E** spectral pattern with the change of the rotor spinning rate (see Figure S1). We assumed that this effect could be due to two factors, the change in temperature and/or the power of the centrifuge. The spinning of the NMR rotor causes heating through air friction. The temperature of the sample increases rapidly as the spinning speed increases. We observed up to a 15 °C factor at 13 kHz spinning speed compared to only ca. 5 °C at 8 kHz from ambient temperature using a 4 mm probe head. These values are consistent with the previously published data.^51,52^

According to the X-ray data-based structure, the **SM_E** form contains water molecules in the crystal lattice (see X-ray Determination of the SM_E Single Crystal X-ray Structure). It is known that in some cases, weakly bound crystallographic water can be removed from the lattice with increasing temperature. Such a thermal effect could be a possible explanation for the difference between the structure of **SM_E** and that of the Xadago tablet.

**Figure 8:**
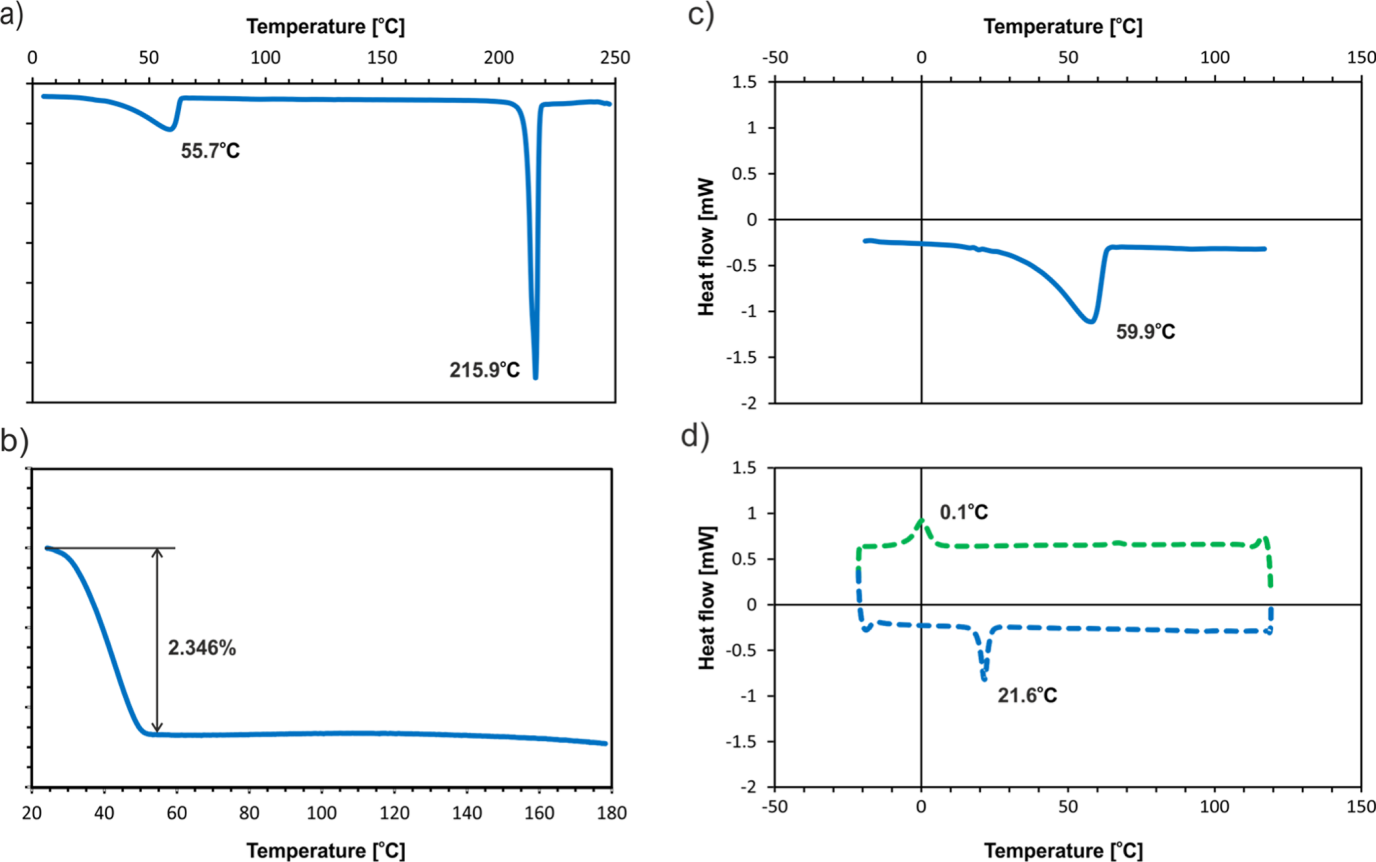
DSC (a) and TGA (b) plots for the **SM_E** sample with the heating rate of 5 °C min^–1^. Additional DSC plots using a new portion of the sample during (c) first heating and (d) first cooling and second heating runs in the range of temperatures below the melting point with the rate of 5 °C min^–1^.

To examine, we performed a detailed DSC and TGA study of the **SM_E** sample (Figure 8). In the first stage, thermal stability was tested by DSC in the temperature range between 0 and 250 °C (Figure 8a). The DSC profile shows two endothermic peaks. The strongest one at 215.9 °C can be easily assigned to the melting process of the material (according to the literature data, mp 208–212 °C).^88^ Much more interesting in the context of this study is the broad endothermic peak with a maximum at 55.7 °C. The energetic effect is quite significant and suggests an important reorganization of the phase. The TGA analysis (Figure 8b) shows a loss of 2.346% total weight around 30–60 °C. The observed value is very close to the theoretical content of water in the **SM_E** (2.211%). Such an agreement clearly suggests that the discussed phase transition could be assigned to the loss of one molecule of water from the crystal lattice. Usually, a good practice to apply in such cases is to check the observed effect in a new portion of the sample by performing multiple heating-cooling runs in the range of temperatures below the melting point (Figure 8c,d). Since the thermal effect was attributed to the dehydration process, it should be only observed during the first heating run (Figure 8c). However, after the first run, another, very subtle thermal process is registered. The observed transitions are fully repetitive through multiple heating-cooling procedures (see Figure S2 for the additional heating-cooling curve). Both peaks at around 21 °C (while heating) and around 0 °C (while cooling) have the same very weak thermal effect of ca. 6.8 J/g and represent a fully reversible phase transition. The 20 °C difference in the position of the thermal transformation while cooling and heating is a well-known effect of shifting of the peak position by the thermal gradients in the sample.^89^ A very similar observation, though without a detailed explanation, was reported in the patent claim mentioned earlier in the text.^12^

Keeping in mind the significant evidence of a dehydration process of **SM_E**, we prepared a new sample called **SM_D** by heating the starting material **SM_E** at 80 °C for 1 h in an oven. For both samples, we performed an elemental analysis of the total carbon, hydrogen, and nitrogen contents. This analysis also supported the loss of one molecule of water while the **SM_E** sample is heated. Just for testing purposes, we verified if **SM_E** undergoes the same transformation when it is kept over P_2_O_5_ in a desiccator for a week. It was also possible to transform the **SM_D** form to **SM_E** by placing the **SM_D** in an open Petri dish and keeping it without a direct solvent-sample contact for 14 days in a diffusion vessel filled with water. These simple procedures confirmed the reversibility of the dehydration/hydration process. It is worth noting that very similar rehydration processes were observed in the case of Xadago when a mechanically damaged tablet was stored in a humid environment (see Figure S3). This means that such unexpected events can occur even with commercial drugs during storage.

**Figure 9:**
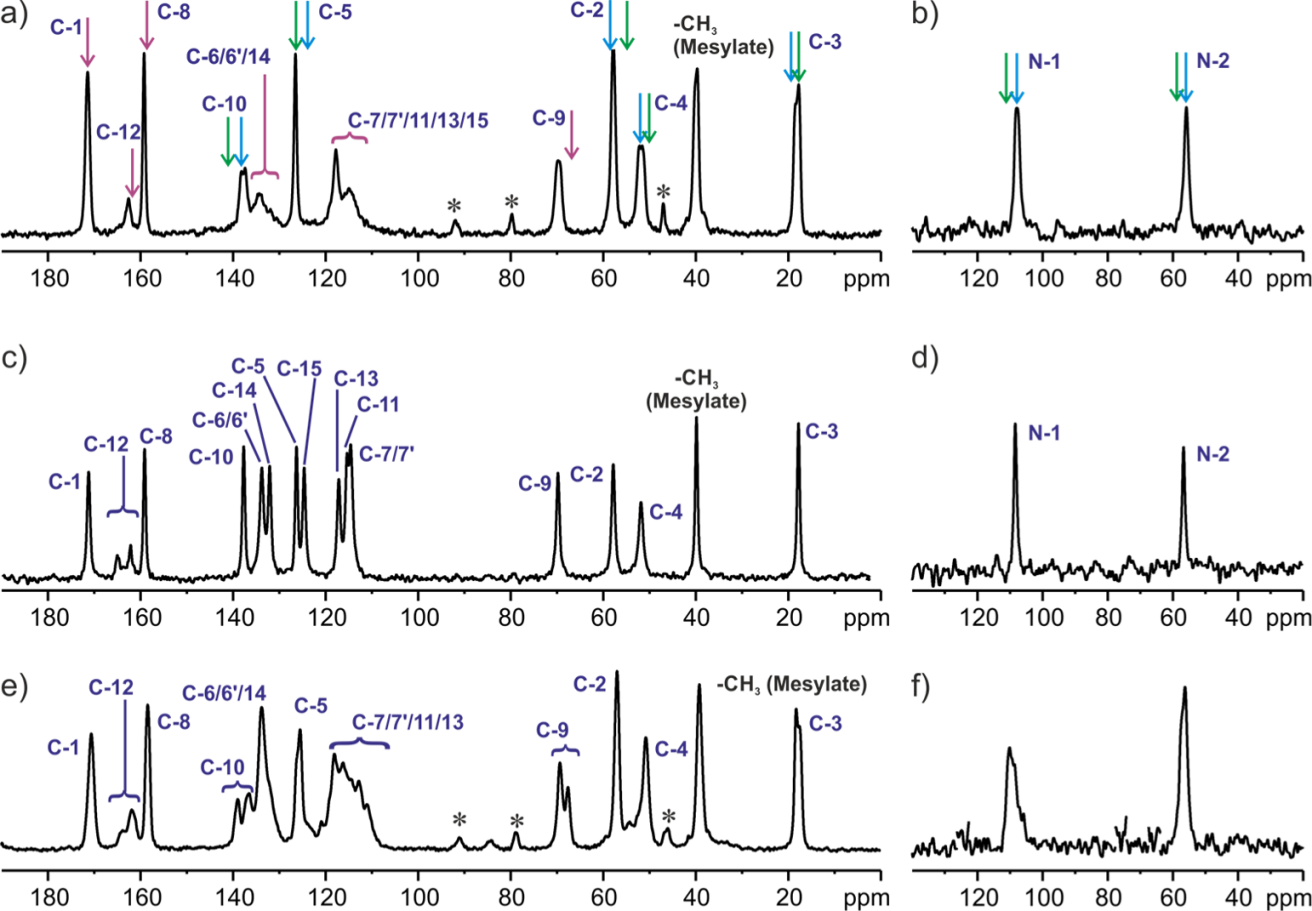
^13^C and ^15^N CP MAS NMR spectra of **SM_D** at ambient temperature (a,b), at 50 °C (c,d), and at −30 °C (e,f) recorded at a spinning rate of 8 kHz and a ^1^H Larmor frequency of 400.1 MHz. The blue (molecule “A”), green (molecule “B”). and purple (overlapped) arrows indicate positions of resonances for **SM_E** (a,b). The assignment of signals (c–e) is shown according to the results from Thermal Transformations of Sample SM_E and Determination of SM_D Crystal Structure Based on the Data Obtained in a PXRD Measurement and Its Validation Using Advanced Solid-State NMR and GIPAW Calculations. Asterisks indicated spinning sidebands.

The first examination of the obtained material (**SM_D**) was performed by ^13^C as well as ^15^N CP MAS spectroscopies at ambient temperature (Figure 9a,b). The difference in relation to ^13^C and ^15^N CP MAS for **SM_E** is clear (see the Figure 6). At a first glance, it is obvious that the characteristic doubling of NMR peaks for **SM_E** (*Z*′ = 2 structure) is gone or at least significantly reduced in the case of the **SM_D** structure. It suggests that the number of safinamide (as well as mesylate) molecules in the asymmetric part of the unit cell may have changed. Additionally, most of the solid-state NMR resonances overlap with the resonances observed for molecule “A” in **SM_E** (blue arrows). It is not surprising because molecule “A” in contrast to molecule “B” does not have any hydrogen bonds with the water molecule which was discussed *supra*. A thought-provoking observation is also the very low intensity and broadening of CH signals in the aromatic region. Trying to explain the nature of these ambiguities, we varied the setup in the CP MAS experiment. Unfortunately, despite several attempts, no spectral improvement was observed. Another important feature of the ^13^C CP MAS results is the straightforward evidence about the equivalence between **SM_D** and the **SM** polymorph originally found in the Xadago tablet (see Figures 2b and 9a).

Taking into account the fact that the DSC measurements of **SM_D** indicate a reversible phase transition very close to room temperature, it seems to be important to record the NMR spectra above and below this point to see whether change of temperature influences the shape of NMR signals. Figure 9c–f depicts the spectra of **SM_D** recorded at 50 °C as well as at −30 °C. Both of them are significantly different from the room-temperature results (Figure 9a,b) which have to be carefully examined.

Typically, thermal processes affect the intensity and/or broadening of NMR signals. However, in our case, the ^13^C CP MAS **SM_D** spectrum recorded at −30 °C also shows changes in the position of the signals compared to the spectrum recorded at higher temperatures (Figure 9a–d). This is most evident for the C-9 position, where two distinct singularities suggest a crystallographic system with the *Z*′ value higher than 1. In addition, the relative intensities of the ^13^C CP MAS peaks for position C-9 appear to be different from 1:1, which additionally excludes the possibility that *Z*′ is equal to an even number. All of that is also supported by the ^15^N CP MAS spectrum (Figure 9f), where we observed “ragged” rather than clean and smooth shapes of the signals, especially for N-1. It would be very hard to explain all of these effects assuming that *Z*′ is equal to 1 or 2. This problem will be discussed in chapter *v* employing the PXRD technique.

Since the intensity and shape of ^13^C CP MAS CH aromatic signals are changed significantly in the temperature range of −30 to 50 °C (Figure 9), it prompted us to analyze the molecular dynamics of both **SM_D**^**LT**^ as well as **SM_D** forms. Molecular motion on various time scales can be easily probed by solid-state NMR spectroscopy. Here, we applied the 2D PISEMA MAS experiment.^37−39^ This is a well-established solid-state NMR method to measure ^13^C–^1^H dipolar couplings and to study dynamic processes on the kHz time scale.^90,91^ The 2D PISEMA MAS spectra record the dipolar coupling between the specific carbon and the closely located protons. According to the equation *D* = −(μ_0_ℏ/8π^2^)(γ_*i*_γ_*j*_)/*r*_*ij*_^3^, the dipolar coupling constant for a typical ^13^C–^1^H distance equal to 1.09 Å is 23.3 kHz. The experimentally measured splitting values are lower than the calculated ones due to the scaling factor which reduced observed splitting.^92^ For the PISEMA MAS NMR experiment, the exact Hartmann–Hahn matching condition gives a scaling factor of 0.577 (cos 54.7°), which gives the expected splitting value of ca. 13.4 kHz (23.3 kHz × 0.577).^37^ Furthermore, since motional processes reduce the observed dipolar coupling value, it can be quantitatively probed by comparing the observed value to the rigid limit.^93−95^

**Figure 10:**
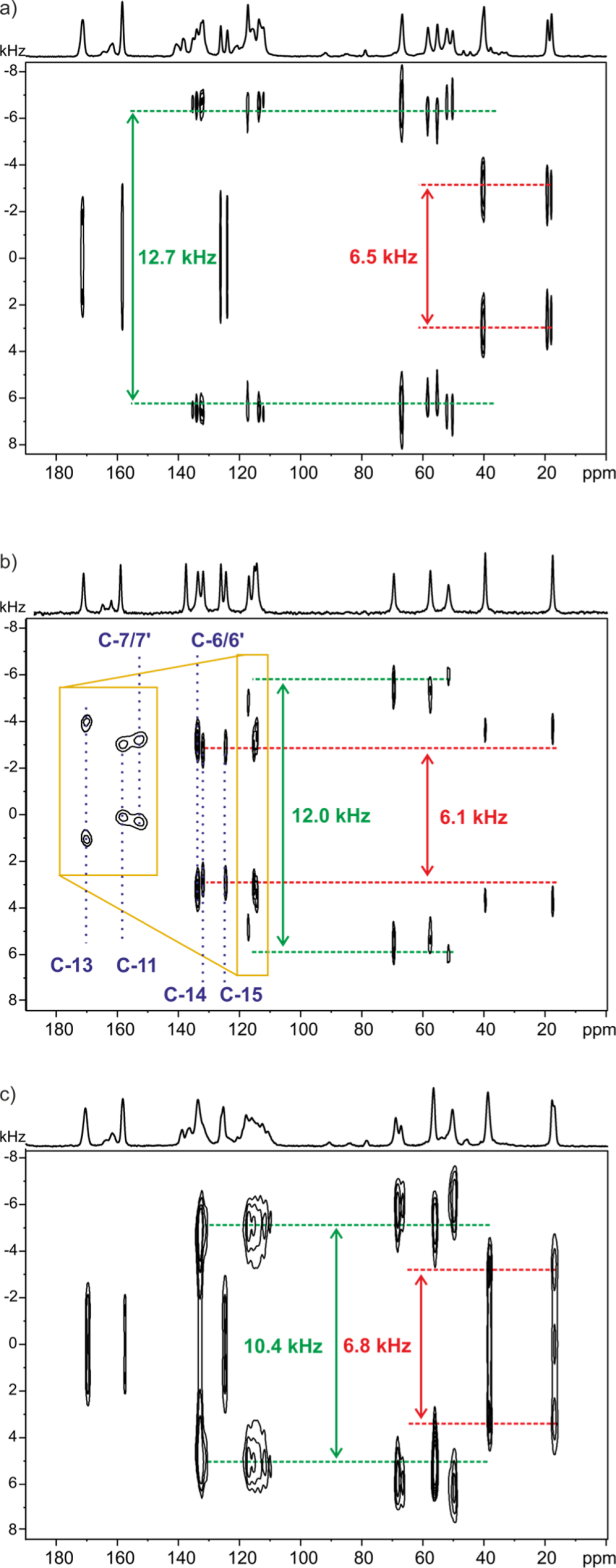
2D PISEMA MAS spectra for samples **SM_E** (a), **SM_D** at 50 °C (b), and **SM_D**^**LT**^ (c). The highest splitting values are labeled in each spectrum. Spectra were acquired at a 13 kHz spinning rate and a ^1^H Larmor frequency of 400.1 MHz.

Figure 10 shows the 2D PISEMA MAS NMR spectra for samples **SM_E** (at ambient temperature), **SM_D** (recorded at 50 °C), and **SM_D**^**LT**^ displayed in the 2D contour plots. In the case of **SM_D**, a short explanation is necessary here. Since, the **SM_D** form exhibits very low intensity aromatic signals at room temperature, it was not possible to perform a dynamic analysis with confidence at this point. Therefore, for the purpose of the 2D PISEMA MAS NMR experiment, the sample was heated up to 50 °C to be above the coalescence temperature. As it is easily seen in Figure 10, the dehydration process made significant changes in the matter of dynamic behaviors. The starting material (**SM_E**) can be assigned as the rigid system (except the −CH_3_ groups). It is consistent with our previous observation reported in PXRD and Solid-State NMR Analysis of Xadago Tablet where we concluded the presence of a static (rather than dynamic) disorder for the fluorophenyl group in molecule “B.” The dehydrated polymorphs of **SM** present much higher flexibility than the hydrated form of **SM**. The spectrum for **SM_D** at 50 °C (Figure 10b) proves without any doubt the presence of molecular dynamics of both aromatic parts of the molecule. It means the movement of the aromatic rings is not blocked as it was happening for **SM_E**.^93,95^ Additionally, the fact that we observe a slightly higher dipolar coupling value for C-6/6′/7/7′ (7.0 kHz) than for C-11/14/15 (6.1 kHz) of CH aromatic resonances is very interesting. It suggests a different topology of the dynamic process for both rings. According to our previous study, we can assign the latter value to the 180° ring flip, while the higher value observed for the fluorophenyl ring corresponds to the slightly smaller topological movement as the wobbling with lower than 180° amplitude.^75,96^ Interestingly, the ^13^C–^1^H dipolar coupling for the C-13 position, which also belongs to the part of the molecule affected by the dynamic process, has the value very close to the rigid limit. It can be easily explained by the fact that C-13 is located directly on the rotation axis of the fluorophenyl ring and thus does not undergo molecular motion. All of these observations nicely support the assignments of aromatic carbon signals primarily presented in Figure 9c. Unfortunately, due to the very broad peaks in the aromatic region of **SM_D**^**LT**^, it is difficult to discuss its dynamic processes in detail. However, the average dipolar coupling value of ca. 10.4 kHz allows to assign it as a low-amplitude wobbling of aromatic rings.^75,93,95−97^

### Determination of SM_D Crystal Structure Based on the Data Obtained in a PXRD Measurement and Its Validation Using Advanced Solid-State NMR and GIPAW Calculations

**Figure 11:**
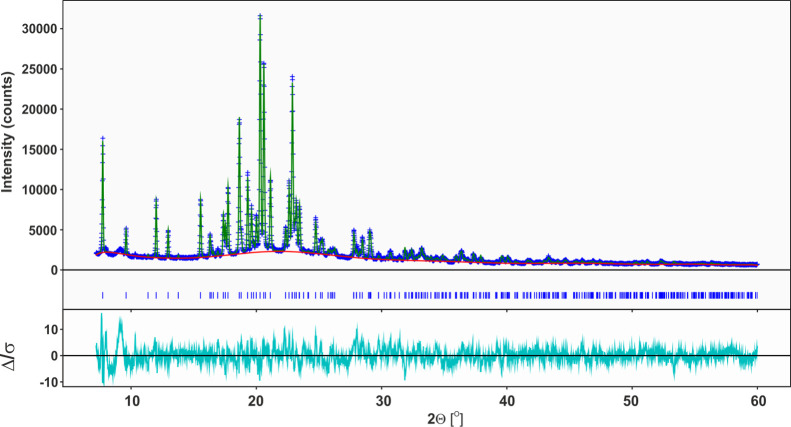
Rietveld curves for **SM_D**.

**Figure 12:**
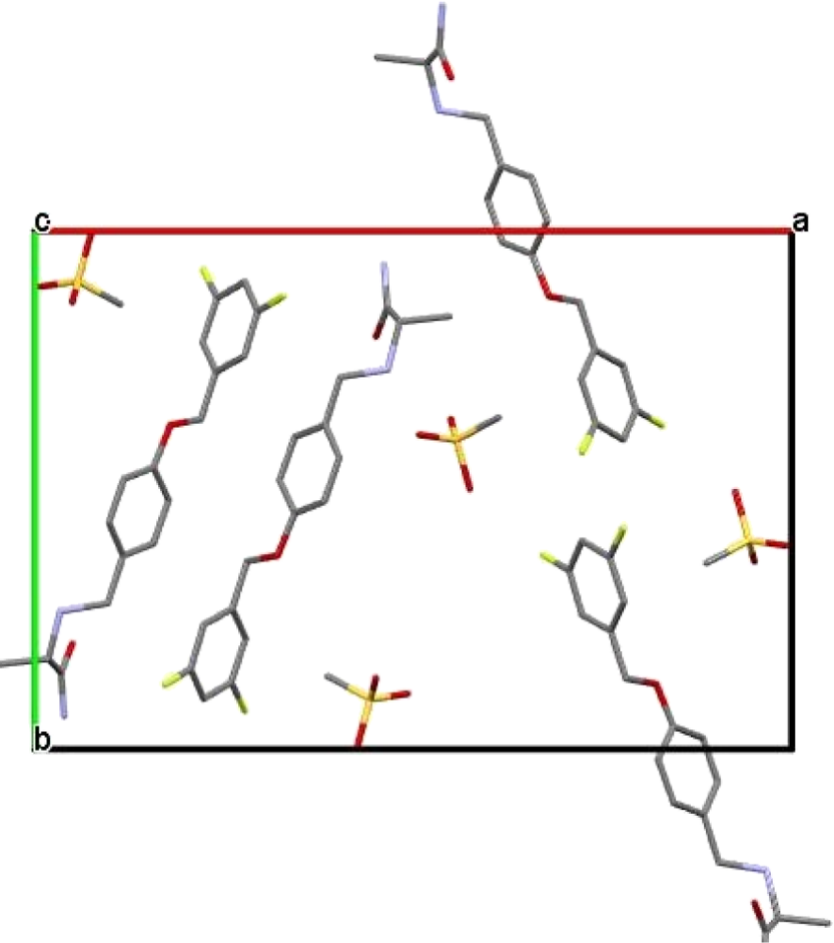
PXRD unit cell of **SM_D** polymorph displayed along the “*c*” direction.

The material obtained after thermal treatment did not allow single-crystal X-ray measurements to be performed because it was not possible to select a crystal of adequate quality for that purpose. Since it is a very frequent situation when the desolvation process causes changes in the morphology of crystallites eliminating the possibility of single-crystal X-ray measurement, we also made an attempt to obtain monocrystalline **SM_D** material by crystallization procedures from various solvents. The motivation for the search of optimal crystallization conditions was the general information in the patent claim that good quality crystals of the anhydrous form of **SM** can be obtained.^12^ Unfortunately, despite long and intense efforts, our attempt concluded without a success. Therefore, the application of NMR crystallography was the best choice to determine the crystal structure. First, we applied PXRD methodology. The experimental powder diffraction data for **SM_D** were successfully indexed in an orthorhombic cell by applying the successive dichotomy method DICVOL04 as implemented in Expo2014 software.^29−31^ The obtained cell parameters accounted for all but three small impurity lines among the observed diffraction peaks. Despite numerous attempts to find alternative indexing that would include these lines, no better solution was detected. Based on the systematic absence analysis and Le Bail fitting,^98^ the space group *P*2_1_2_1_2_1_ was selected with the *R*_wp_ value equal to 5.75%. The model of the crystal structure was generated using the global optimization technique implemented in FOX.^32,33^Figure 11 presents the Rietveld refinement result for **SM_D** diffractogram by utilizing GSAS-II software.^34^ In the Rietveld refinement, standard restraints were applied to bond lengths and angles, as well as planar restraints to the aromatic parts of the molecule. Since the molecular dynamic investigation (Thermal Transformations of Sample SM_E) suggests molecular motion of aromatic rings, the fractional occupation of the fluorine atom in two positions was applied. Finally, the structure model gave a satisfactory fit to the measured diffraction data, which is reflected in the difference curve (Δ/σ) and the *R*_wp_ = 6.70% value. The broad reflex at 9.7° which makes the highest Δ/σ value is an artifact of the measurement setup, and it was not taken into account during the analysis. The obtained crystal structure solution is attached to the Supporting Information as a crystallographic information file (cif). The crystallographic details as well as the unit cell view are shown in Table 2 and Figure 12 respectively. The technical details are presented in the Experimental Section.

**Table 2:** Crystallographic Details Obtained from the PXRD Analysis of SM_D

empirical formula	C_18_FN_2_O_5_S
dormula weight	375.27
*a* (Å)	22.7568(3)
*b* [Å)	15.5428(4)
*c* [Å)	5.55510(10)
α (deg)	90
β [deg]	90
γ [deg]	90
*V* [Å^3^]	1964.86(5)
*Z*	4
*Z*′	1
radiation wavelength [Å]	1.5418
space group	*P*2_1_2_1_2_1_ (19)
*R*_wp_	6.70
density (calc) [g/cm^3^]	1.2686

It has to be stressed that the PXRD patterns for **SM_D** explain most of the observed PXRD reflexes for the Xadago tablet (Figure 2a). It is consistent with our solid-state NMR results and confirms that **SM_D** polymorph is the commercial form of **SM**. Based on the PXRD reflex positions, we identified our **SM_D** form as the **A1** reported in the patent claim mentioned earlier.^12^

In principle, the NMR Crystallography workflow requires at least rough atomic coordinates as the starting point to proceed to the computational stage. The PXRD crystal structure solution result usually is characterized by sufficient precision for DFT-D calculations. Preceding the calculations of the NMR parameters (important for final validation of the structure), geometry optimization allowing the variation of all atomic positions was performed. The final DFT-D optimized structure is only slightly different compared to the starting PXRD model. If we superimpose both crystal structures, we can see that the RMSD among equivalent atomic positions is as low as 0.5 Å for clusters containing 30 molecules (see Figure S4).

The final validation of the DFT-D structure was made with the help of solid-state NMR methodology. Although simple 1D solid-state NMR spectra allow a quick distinction between all of the discussed **SM** forms through the fingerprint of a specific material, they do not allow to obtain enough structural constraints to judge the atomic scale arrangement. Therefore, the validation of the **SM_D** form needed a more robust technique such as the fast MAS NMR, allowing for spinning 1.3 mm rotors up to 67 kHz. There are several advantages of such a method. For example, it allows a significant reduction in the amount of required sample for the measurement, and there is a possibility to record ^1^H spectra with lower broadening of signals or the option to apply inverse detected pulse sequences and acquire 2D solid-state NMR correlations. Unfortunately, fast MAS application, besides a lot of tremendous advantages, carries some drawbacks. The extremely high spinning rate of the sample significantly increases the heating of the rotor through air friction up to a factor of 60 °C. Moreover, the centrifuge power is extremely high which can further accelerate the phase transitions. It is worth mentioning that, despite several attempts, we were not able to run measurements for **SM_E** as well as **SM_D**^**LT**^ employing the very fast MAS technique. The application of a cooling system combined with the relatively small spinning frequency (40 kHz) failed and did not prevent the change of the **SM_E** and **SM_D**^**LT**^ to **SM_D** sample during the measurement. In the end, we were able to perform the fast MAS measurements for **SM_D** form only.

**Figure 13:**
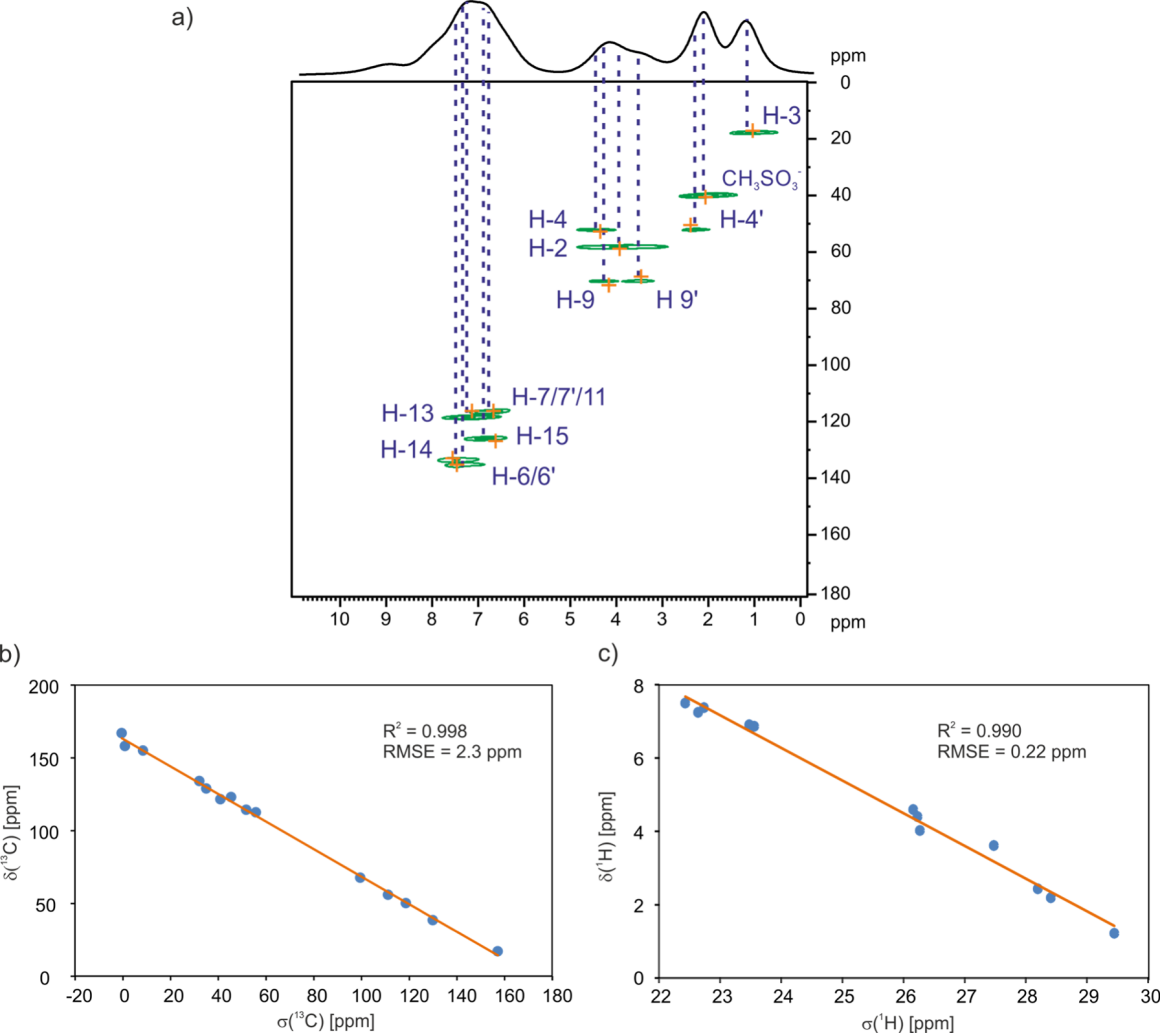
(a) ^13^C–^1^H invHETCOR MAS NMR spectra of **SM_D** recorded at 90 °C with a spinning rate of 60 kHz at a ^1^H Larmor frequency of 600.1 MHz with a second ^13^C → ^1^H CP contact time of 100 μs. A one-pulse ^1^H MAS spectrum is shown at the top. The orange crosses represent GIPAW-calculated NMR correlations for C···H distances up to 1.5 Å. Isotropic ^13^C (a) and ^1^H (b) NMR values correlation (experimental chemical shifts vs GIPAW nuclear shieldings) for **SM_D**.

From NMR spectroscopy point of view, the ^1^H nucleus is the most sensitive probe for studying the local structure and remote contacts. Unfortunately, the measurement and assignment of signals in ^1^H solid-state NMR spectra are incomparably more difficult than for a ^13^C nucleus. Whereas proton NMR experiments are the most routine measurements in the liquid state, their solid-state equivalent still is challenging. The main problem with ^1^H solid-state NMR spectra for organic solids at natural abundance is the low signal resolution due to the extremely strong homogeneous ^1^H–^1^H dipolar couplings. Fortunately, outstanding developments in fast MAS probes significantly reduce the broadening of NMR signals. In particular, it allows measuring of 2D heteronuclear experiments with indirect inverse (inv) observation via ^1^H such as a ^13^C–^1^H invHETCOR MAS NMR experiment. Figure 13a shows the ^13^C–^1^H invHETCOR MAS spectra acquired with a short (100 μs) ^13^C → ^1^H CP contact time such that only cross peaks corresponding to short C···H distances, mostly direct C–H bonds, are observed. The ^1^H–^13^C invHETCOR provided ^1^H chemical shifts which can be used for final validation of the DFT-D **SM_D** structure solution. As we have shown in previous section, the correlation between the calculated and experimental ^13^C chemical shifts can be used as a method of verifying the quality of the structure refinement. ^1^H nucleus, as shown for the **SM_E** sample, is even more sensitive to the local arrangement of atoms in the crystal lattice.^72,82−86^ In that case, for the PXRD-based structure solutions, where the accuracy is much lower than for single-crystal-based X-ray diffraction methods, it is preferred to validate the obtained crystallographic data not only by ^13^C but also with the assistance of ^1^H chemical shifts. Figure 13b,c shows the correlation between calculated (GIPAW) and experimental NMR parameters. The RMSE values of 0.22 and 2.3 ppm for ^1^H and ^13^C, respectively, (Table S2) represent a very good agreement between experimental and GIPAW-calculated chemical shifts.^72,82−86^ The obtained correlations support the correctness of the proposed structural solution.

### Determination of **SM_D**^**LT**^ Crystal Structure Based on the PXRD Measurements and Its Relation to the **SM_D** Structure

**Figure 14:**
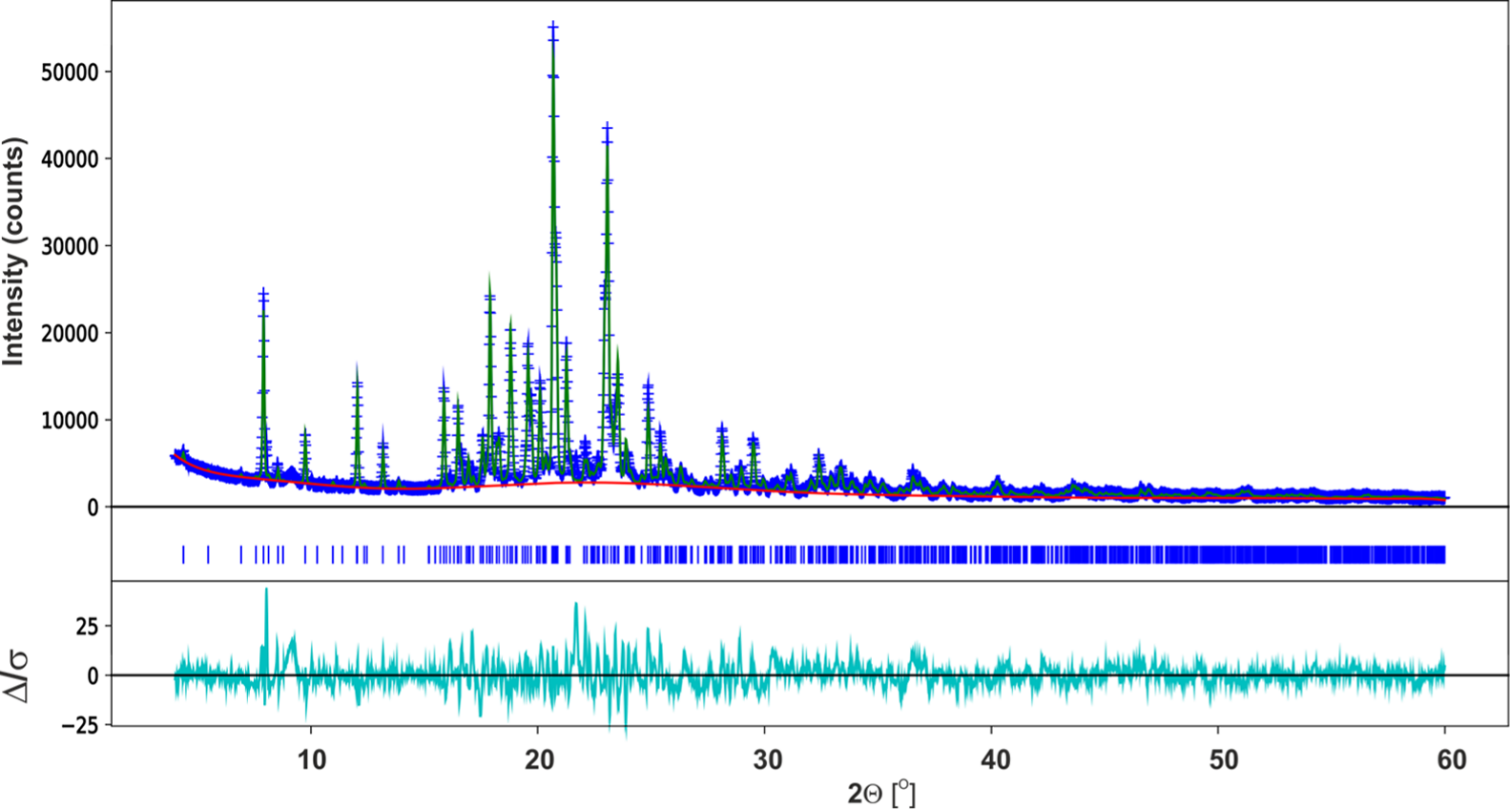
Rietveld curves for **SM_D**^**LT**^.

Analogous treatment with PXRD-related methods to the one used for **SM_D** was applied for **SM_D**^**LT**^. The obtained Le Bail fitting with the *R*_wp_ value equal to 7.05% gave the same crystal symmetry as for **SM_D** with very similar unit cell parameters, except for vector b which was approximately 3 times longer than for **SM_D**. It clearly suggests that the structure is *Z*′ = 3. It is also justified to assume that the molecular packing is not very different between **SM_D** and **SM_D**^**LT**^. It is also consistent with the ^13^C CP MAS spectra (Figure 9e) of **SM_D**^**LT**^ suggesting *Z*′ > 2 type of structure with very similar peak positions to the **SM_D** (Figure 9c). Taking all of the above into account, our structural model was refined in a similar workflow as presented in Determination of SM_D Crystal Structure Based on the Data Obtained in a PXRD Measurement and Its Validation Using Advanced Solid-State NMR and GIPAW Calculations. The final Rietveld fit is shown in Figure 14 with its difference curve (Δ/σ). The obtained *R*_wp_ = 9.37% is a bit worse than for **SM_D**. However, when we consider that **SM_D**^**LT**^ is a much larger system than **SM_D**, the obtained value is still reasonable. The crystallographic details are shown in Table 3.

**Table 3:** Crystallographic Details Obtained from the PXRD Analysis of **SM_D**^**LT**^

empirical formula	C_18_FN_2_O_5_S
formula weight	375.27
*a* [Å]	5.52277(25)
*b* [Å]	46.5689(27)
*c* [Å]	22.3161(11)
α [deg]	90
β [deg]	90
γ [deg]	90
*V* [Å3]	5739.5(7)
*Z*	12
*Z*′	3
radiation wavelength [Å]	1.5418
space group	*P*2_1_2_1_2_1_ (19)
*R*_wp_	9.37
density (calc) [g/cm^3^]	1.3029

**Figure 15:**
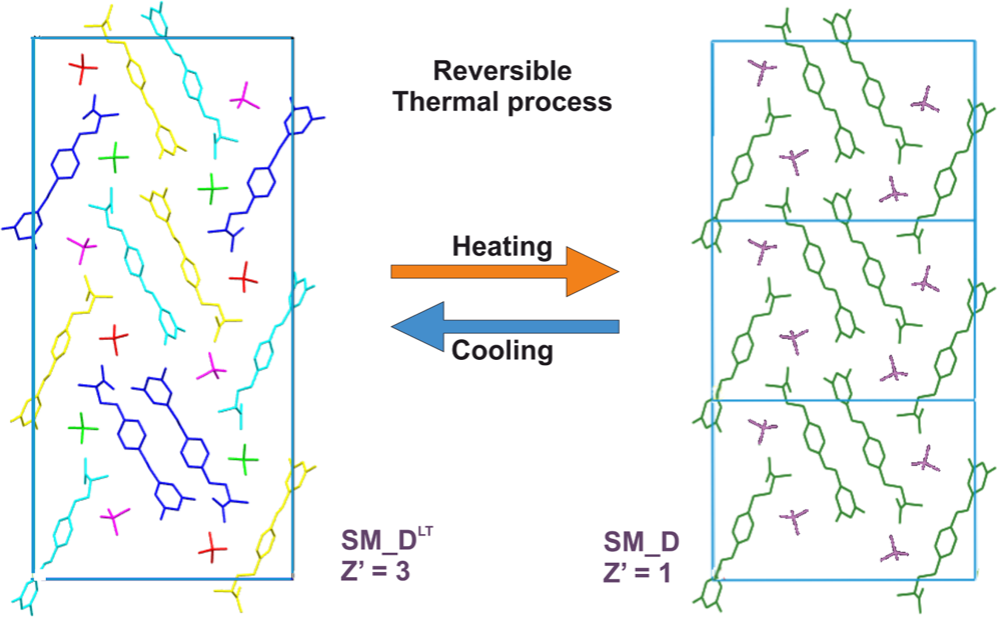
Relation *Z*′ = 1 ↔ *Z*′ = 3 between PXRD unit cells of **SM_D**^**LT**^ and **SM_D**. Molecules are colored according to the symmetry equivalence and shown without hydrogens.

**Figure 16:**
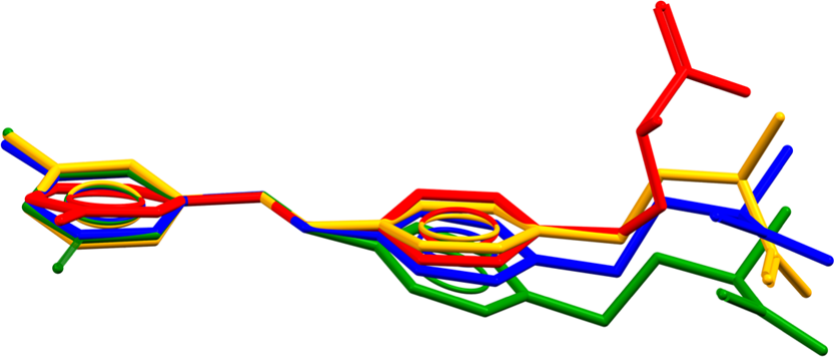
Superposition of nonequivalent molecules in **SM_D**^**LT**^ (green, blue, and red) and **SM_D** (orange).

This nondestructive transformation fits well to the already available literature showing the great tendency of safinamide derivatives for creating various temperature-dependent polymorphic forms.^13^ Taking into account all facts above, it is obvious that the reversible transformation **SM_D**^**LT**^ ↔ **SM_D** is connected with the *Z*′ = 3 ↔ *Z*′ = 1 change that is pictographically shown in Figure 15. The most probable explanation of this phase change is that while **SM_D** form is cooled, molecular dynamic is slowing down what causes the formation of three slightly different conformers of safinamide (Figure 16). In this way, the system reduces its symmetry in the crystal lattice and has to be described by a unit cell 3 times as large as the one observed for **SM_D**.

Our observations for **SM_D** are consistent with the conclusions described by Nangia who showed that polymorphs with a larger number of symmetry-independent molecules (high *Z*′) generally led to the occurrence of polymorphism when compared with the polymorphs with lower *Z*′ values.^99^ The **SM_D** case confirms that organic molecules with flexible torsions and low-energy conformers have a greater likelihood of exhibiting conformational polymorphism.^100^

## Conclusions

In this work, we showed how complex and nonobvious processes can be observed in the crystal lattices of compounds that are used as commercial drugs. When examining Xadago tablets, which contain **SM** as the API, we noticed several processes, each of which is reversible. The first is hydration and dehydration which is controlled by the temperature and humidity of the environment. The freshly crystallized **SM** employing a mixture of solvents water/ethanol (vol/vol 1:9) forms a hemihydrate (orthorhombic system with the *P*2_1_2_1_2_1_ space group). At moderate temperature (60–80 °C), the crystals lose water creating an anhydrous form with *P*2_1_ space groups. This process can be reversed when the sample is stored in a humid environment. The anhydrous form undergoes thermal phase transition forming different polymorphs **SM_D**^**LT**^ (*Z*′ = 3) ↔ **SM_D** (*Z*′ = 1). This polymorphic alternation takes place in the temperature range 0–20 °C which is the typical temperature for drug storage. Studying the correlation of structural changes in MS with the therapeutic properties of a drug is beyond the scope of our work. However, it seems apparent that such effects must be considered when formulating and storing a drug.

## Supporting Information Available

^13^C CP MAS NMR spectra recorded at a spinning rate of 8 and 13 kHz, additional DSC plots, results for the Xadago drug when a mechanically damaged tablet was stored in a humid environment at room temperature, superposition of the PXRD crystal structure solution prior and after DFT-D geometry optimization, and tables containing the experimental chemical shifts and GIPAW-calculated NMR shieldings (PDF)
Crystallographic information for the crystal structures solved by the PXRD methodology (ZIP)
