# Supplementary material for: Solid-State Study of the Structure, Dynamics, and Thermal Processes of Safinamide Mesylate—A New Generation Drug for the Treatment of Neurodegenerative Diseases
Source: Mol Pharm. 2021 Dec 3;19(1):287–302. doi: 10.1021/acs.molpharmaceut.1c00779 (PMC8728732; doi:10.1021/acs.molpharmaceut.1c00779)
Supplement: Supplementary file 1 — mp1c00779_si_001.pdf [file mp1c00779_si_001.pdf]

Solid-State Study of the Structure, Dynamics and Thermal Processes of Safinamide Mesylate - a New Generation Drug for the Treatment of Neurodegenerative Diseases.

Tomasz Pawlak,*[1] Marcin Oszajca,[2] Małgorzata Szczesio[3] and Marek J. Potrzebowski[1]

[1] Polish Academy of Sciences, Centre of Molecular and Macromolecular Studies, Sienkiewicza 112, 90-363 Lodz, Poland.

[2] Faculty of Chemistry, Jagiellonian University, Gronostajowa 2, 30-387 Krakow, Poland

[3] Institute of General and Ecological Chemistry, Faculty of Chemistry, Lodz University of Technology, Żeromskiego 116, 90-924, Lodz, Poland

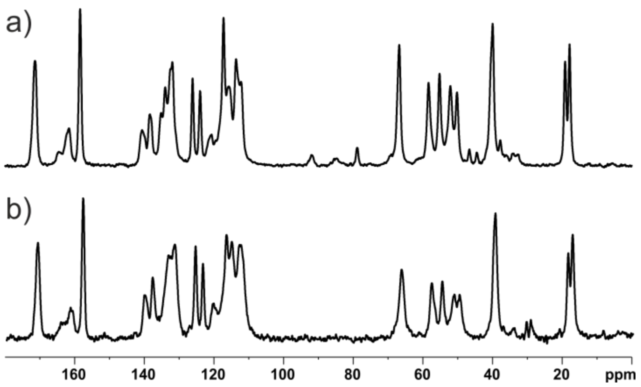

Figure S1. ^{13}C CP MAS NMR spectra of **SM_E** recorded at a spinning rate of 8 (a) and 13 kHz (b) at ambient temperature.

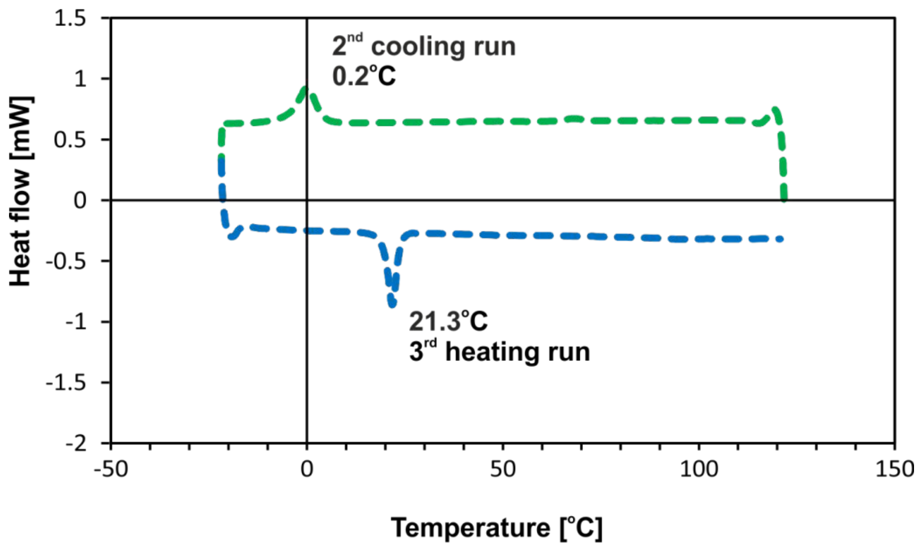

Figure S2. The DSC plot for **SM_E** sample while 2nd cooling follow by 3rd heating run in the in the range of temperatures below the melting point with the rate of 5 °C min^{-1}.

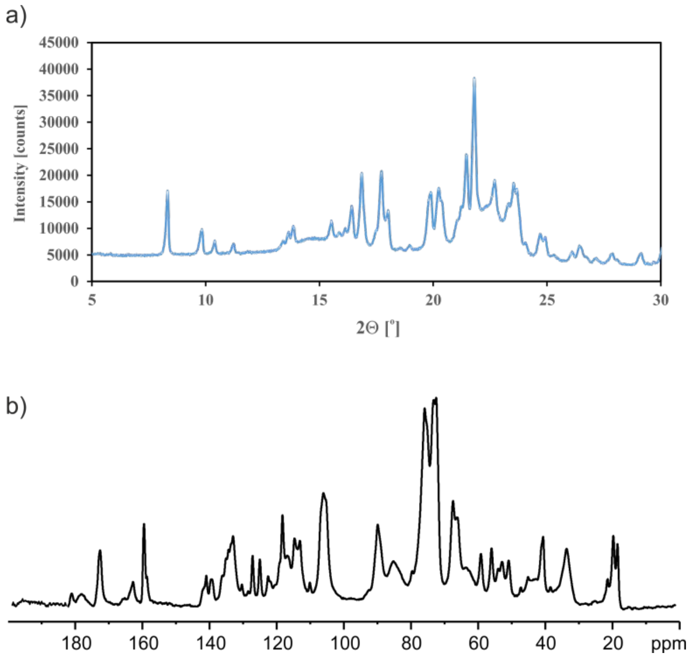

Figure S3. The results for Xadago® drug when a mechanically damaged tablet was stored in a humid environment at room temperature: a) X-Ray powder pattern recorded in Bragg Brentano geometry with a Cu Kα (λ = 1.5425 Å) source. b) ^{13}C CP MAS NMR spectrum recorded at a spinning rate of 8 kHz and 14 tesla, corresponding to a ^{1}H Larmor frequency of 600.1 MHz. The recycle delay was 30 s.

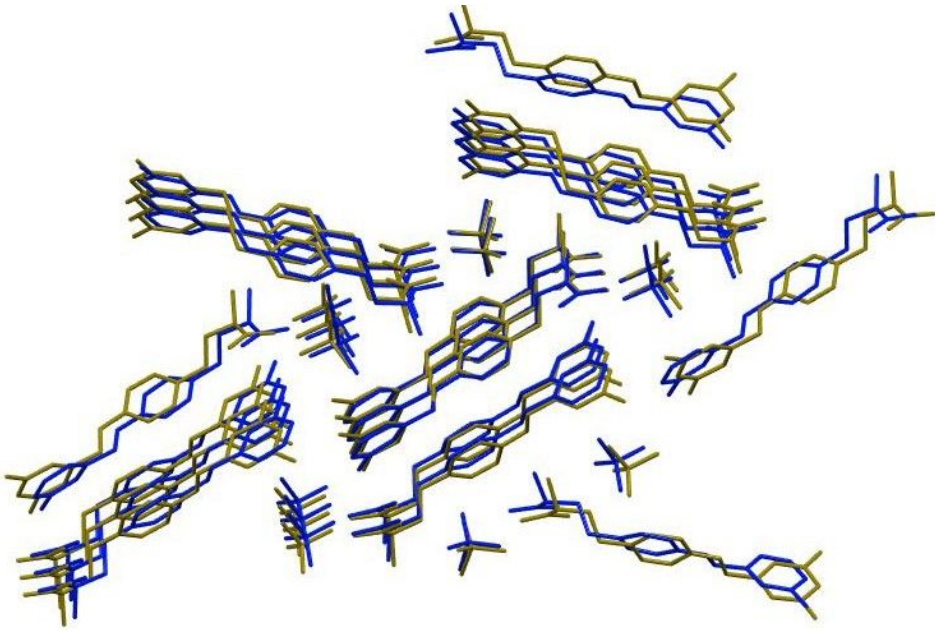

Figure S4. Superposition of PXRD crystal structure solution of **SM_D** prior (blue) and after (yellow) DFT-D geometry optimization.

Table S1. ^{13}C NMR experimental chemical shifts (δ) and GIPAW calculated nuclear shieldings (σ) [in ppm] (after DFT-D geometry optimization allowing the unit cell parameters to vary) for **SM_E**.

**Structure (position)**	**δ(^{13}C) SM_E** (molecule A) Exp	**σ(^{13}C) SM_E** (molecule A) GIPAW	**δ(^{13}C) SM_E** (molecule A) GIPAW	**δ(^{13}C) SM_E** (molecule B) Exp	**σ(^{13}C) SM_E** (molecule B) GIPAW	**δ(^{13}C) SM_E** (molecule B) GIPAW
**C-1**	171.6	-0.5	168.5	171.6	0.4	167.6
**C-2**	58.5	112.2	59.1	55.3	117.1	54.3
**C-3**	19.2	153.9	18.6	17.9	155.2	17.3
**C-4**	52.1	120.5	51.0	50.3	123.3	48.3
**C-5**	124.1	47.7	121.7	126.3	44.0	125.3
**C-6**	132.6	37.1	132.0	132.6	34.6	134.4
**C-6'**	132.6	33.2	135.8	132.6	37.1	132.0
**C-7**	117.3	53.8	115.8	117.3	57.8	111.9
**C-7'**	113.1	57.8	111.9	113.1	53.5	116.1
**C-8**	158.3	10.4	157.9	158.3	10.4	157.9
**C-9**	66.9	102.3	68.7	66.9	102.7	68.3
**C-10**	138.3	31.6	137.3	140.6	28.1	140.7
**C-11**	113.1	56.7	112.9	113.1	56.7	112.9
**C-12**	163.1	1.9	166.1	163.1	1.6	166.4
**C-13**	113.1	53.8	115.8	113.1	54.8	114.8
**C-14**	132.6	34.8	134.2	132.6	36.2	132.8
**C-15**	117.3	53.2	116.3	117.3	51.0	118.5
**CH_3SO_3-**	40.0	129.2	42.6	40.0	131.5	40.3

Table S2. ^{1}H and ^{13}C NMR experimental chemical shifts (δ) and GIPAW calculated nuclear shieldings (σ) [in ppm] (after DFT-D geometry optimization allowing the unit cell parameters to vary) for **SM_D**.

Structure (position)	δ SM_D Exp	σ SM_D GIPAW	δ SM_D GIPAW
**C-1**	171.0	-0.5	167.5
**C-2**	57.4	111.1	59.3
**C-3**	17.5	157.1	14.6
**C-4**	51.4	118.6	52.0
**C-5**	126.0	45.4	123.0
**C-6[a]**	132.0	35.0	133.1
**C-7[a]**	115.3	55.7	113.0
**C-8**	158.8	8.5	158.8
**C-9**	69.3	99.5	70.5
**C-10**	137.3	32.1	135.9
**C-11**	115.3	52.7	115.9
**C-12**	162.0	0.9	166.2
**C-13**	117.1	51.7	116.9
**C-14**	132.0	36.8	131.3
**C-15**	124.5	40.9	127.4
**$\underline{C}H_3SO_3$-**	39.4	129.9	41.0
**H-2**	4.01	26.27	4.20
**H-3[a]**	1.18	29.45	1.26
**H-4**	4.41	26.16	4.30
**H-4'**	2.19	28.20	2.41
**H-6**	7.64	22.74	7.46
**H-9**	4.34	26.23	4.24
**H-9'**	3.51	27.48	3.08
**H-13**	7.29	22.64	7.55
**$C\underline{H}_3SO_3$-**	2.15	28.41	2.22

[a] The average of the distinct NMR parameters is presented.
